# Application of the Policy Regime Framework to understand COVID-19 policy response in the Southeast U.S.: How RAPID research can provide lessons learned after a public health crisis

**DOI:** 10.3389/fsoc.2022.959553

**Published:** 2022-12-12

**Authors:** Gregory Johnson, Kasen Wally, Janna R. Willoughby, Ryan Williamson, Kathryn Corvey, Mina Becker, Thomas Moorman, Kelly Dunning

**Affiliations:** ^1^College of Forestry, Wildlife, and Environment, Auburn University, Auburn, AL, United States; ^2^Department of Political Science, Auburn University, Auburn, AL, United States; ^3^School of Public Health, University of Alabama at Birmingham, Birmingham, AL, United States

**Keywords:** COVID-19, Policy Regime Framework, Southeastern United States, vulnerable population, policy response

## Abstract

Quick-response research during a time of crisis is important because time-sensitive findings can inform urgent decision-making, even with limited research budgets. This research, a National Science Foundation-funded Rapid Response Research (RAPID), explores the United States (U.S.) government's messaging on science in response to the COVID-19 pandemic, and how this messaging informed policy. Using rapidly emerging secondary data (e.g., policy documents taken from government websites and others), much of which has since been removed or changed, we examined the interactions between governing bodies, non-governmental organizations, and civilian populations in the Southeastern U.S. during the first 2 years of the pandemic. This research helps to better understand how decision-makers at the federal, state, and local levels responded to the pandemic in three states with the lowest vaccine rates and highest levels of poverty, income inequality, and disproportionate impacts borne by people of color in the nation: Alabama, Louisiana, and Mississippi. This study incorporates the Policy Regime Framework to discuss how two foundational concepts (ideas and institutions) helped govern policy implementation during the COVID-19 pandemic. This research fills a significant information gap by providing a better understanding of how policy regimes emerge across multiple levels of government and impact vulnerable populations during times of a public health crisis. We use automated text analysis to make sense of a large quantity of textual data from policy-making agencies. Our case study is the first to use the Policy Regime Framework in conjunction with empirical data, as it emerged, from federal, state, and local governments to analyze the U.S. policy response to COVID-19. We found the U.S. policy response included two distinct messaging periods in the U.S. during the COVID-19 pandemic: pre and post-vaccine. Many messaging data sources (agency websites, public service announcements, etc). have since been changed since we collected them, thus our real-time RAPID research enabled an accurate snapshot of a policy response in a crisis. We also found that there were significant differences in the ways that federal, state, and local governments approached communicating complex ideas to the public in each period. Thus, our RAPID research demonstrates how significant policy regimes are enacted and how messaging from these regimes can impact vulnerable populations.

## Introduction

In December of 2019, a novel coronavirus that would eventually be called SARS CoV2 began infecting people in China's Wuhan Province. Although the initial infection was isolated to only 59 people, this COVID-19-causing virus quickly spread to other areas and countries (Hubbard, 2021), prompting the World Health Organization to declare COVID-19 a pandemic in March 2020. As the virus made its way to the United States, it was met with a largely disjointed response, which has since been widely criticized internationally (Devlin et al., 2021) and domestically (Lewis, 2021). The pressures of the pandemic also exposed an acute weakness in the federal style of healthcare policy implementation, which divides decision-making power between federal, state, and local governing arrangements (Haffajee and Mello, 2020). As of May 2022, there have been more than half a billion confirmed cases of COVID-19 worldwide resulting in more than 6.2 million deaths (World Health Organization, 2021a). As conflicting and politically divisive information emerged from the White House, such as former President Donald Trump's admission that he was downplaying the severity of the virus and his declaration that COVID-19 would “miraculously go away,”^1^ subnational governments (e.g., U.S. state governments) began to take differing approaches to combat the spread of COVID-19, resulting in “a patchwork of responses by state and local governments, divided sharply along partisan lines” (Altman, 2020; Tollefson, 2020). In addition to some of the conflicting and politically divisive information and differing approaches, many of the policy documents and governmental recommendations have been deleted or removed from government websites since the inauguration of President Joseph Biden. This loss of relevant policy documents makes our RAPID research imperative to show how the government responded to COVID-19 during the emergence of the crisis. By documenting impermanent, time-sensitive COVID-19 policy, our research seeks to untangle a complex web of events, using public policy scholarship to explore policy responses to the COVID-19 pandemic in the Southeastern U.S. We argue that understanding why and how policy responses and messaging around those policies happen could provide insight into other types of public health policies, and that without RAPID funding this information can and will be lost, lessening the ability for our society to learn from policy failures and enact changes necessary to not repeat mistakes even as they may be happening in real-time.

Very few Americans have escaped the effects of the COVID-19 pandemic, whether it be *via* illness or lockdowns (Kupferschmidt and Wadman, 2021). Despite widespread vaccine availability in the U.S., several new and highly transmissible strains of COVID-19 (e.g., Delta and Omicron Variants) have swept across the U.S. in 2021 and early 2022 (Katella, 2021). Unfortunately, experts warn that states with large unvaccinated populations are at the greatest risk of becoming “hotspots” for new infections (Darnell, 2021; DeCiccio, 2021; Mitropoulos and Brownstein, 2021). As of early 2022, Alabama, Louisiana, and Mississippi (the states that this paper focuses on) were still among the least vaccinated states in the U.S. (Mayo Clinic, 2022). This vulnerability demonstrates the importance of examining policy regimes in these U.S. states, a gap that this research fills.

Due to the urgency of the pandemic, decision-makers have prioritized rapid implementation of policy, limiting efforts toward deliberate study of how policy responses to COVID-19 have been implemented and vary across three scales of government: federal, state, and local. Using the Policy Regime Framework's insights on ideas and institutions, we analyze *n* = 277 policy documents to trace policy responses to the pandemic across federal, state, and local actors. We advance the Policy Regime Theory, finding that policy ideas (such as the most up-to-date science on COVID-19) and institutions (such as the government agencies responsible for implementing responses) vary significantly by scale of government (e.g., federal, state, or local government). This research's contribution is an original case study of what policy regimes were implemented across scales to respond to the COVID-19 pandemic so as to provide lessons learned in a vulnerable context.

## Case selection: The Southeastern United States

We focus on the Southeastern U.S. because of the devastating toll that COVID-19 has taken on the region. The American Southeast is one of the regions that has fared the worst throughout the pandemic based on rates of infection, death, and testing, with Alabama, Mississippi, and Louisiana ranking among the worst in the nation (Menendian et al., 2020). When viewed alongside data on income inequality and anti-discrimination laws, which are designed to examine how governmental arrangements (i.e., policy regimes) accommodate the needs of marginalized people, researchers found that in states that failed to respond adequately to the COVID-19 pandemic (i.e., Alabama, Louisiana, and Mississippi), elderly people, disabled people, people of color, and people with low-incomes were disproportionately impacted (Menendian et al., 2020; Othering Belonging Institute, 2021).

There are many reasons why Alabama, Louisiana, and Mississippi have lagged in vaccination rates, witnessed accelerating inequality rates, and suffered extensively throughout the pandemic. Much of the Southern U.S. is rural, making access to healthcare more difficult. In addition, minority communities disproportionately face logistical issues regarding access to education and healthcare, public health infrastructure is often underfunded and understaffed, and mistrust in public health institutions remains a concern (Mitropoulos and Brownstein, 2021; Tai et al., 2021). According to the U.S. Census Bureau, in 2019, Mississippi had the highest rate of poverty in the U.S. (19.6%), followed immediately by Louisiana (19%) and closely by Alabama (15.5%) (U.S. Census Bureau, 2020), and these are amplified in some communities by the significant racial poverty gaps that persist for minorities, especially between Black and white populations in the South (Kent, 2020; U.S. Census Bureau, 2021). Because of these and other pressures, COVID-19 disproportionately affects disadvantaged racial and ethnic minority groups in the U.S. (Laurencin and McClinton, 2020; Romano et al., 2021).

While experts agree that vulnerable populations should be better protected, the responsibility to protect vulnerable populations is in the hands of local, state, and federal governing arrangements, which have at times floundered in the wake of the pandemic. Examples include President Trump's downplaying of the virus (Wolfe and Dale, 2020); the Centers for Disease Control and Prevention's (CDC)^2^ initially slow and flawed testing strategy (Cohen, 2020); conflicting guidance regarding preventative measures such as handwashing vs. mask-wearing (Nagler et al., 2020); the decentralized response among federal, state, and local leaders; and healthcare inequalities fueled by structural racism (Bailey et al., 2021; Lewis, 2021).

Research has shown a need to better understand the governmental responses to the COVID-19 pandemic and explore the interactions between governing bodies, non-governmental organizations, and civilian populations. Greer et al. (2020) argue that to assess governmental responses to COVID-19, one should look at pre-existing social policies, the political regime type(s) and formal institutions present, and the governing capacity. Our research aims to contribute to these gaps to better understand how lawmakers responded to the pandemic in these vulnerable locations. In particular, we leverage RAPID funding and the urgent collection and use of policy document data sources (e.g., policy documents published on government websites) to preserve data on what the government was doing to respond to the crisis in real time. This enables a clear-eyed look at what happened, and the beginning of developing lessons learned for future policy responses.

This study incorporates the Policy Regime Framework developed by May and Jochim (2013) to discuss how decision-makers responded to the COVID-19 pandemic in Alabama, Louisiana, and Mississippi. The Policy Regime Framework, at its core, enables researchers to work backward from a significant policy problem, such as the arrival of COVID-19 in the U.S., to evaluate the governing arrangements, otherwise known as policy regimes, that emerged in response. Additionally, the framework identifies the foundational ideas, institutions, and interests that govern the success or failure of policy implementation (Jochim and May, 2010; May and Jochim, 2013). The COVID-19 pandemic spanned thousands of U.S. jurisdictions, impacting virtually every facet of human life beginning in 2020. To narrow our focus, we apply the Policy Regime Framework specifically on policy responses to COVID-19 that were established to conduct science-based messaging in the Southeastern U.S. In other words, how were agencies at different scales communicating science to the public as a necessary precursor to implementing policy compelling behavioral changes like mask wearing.

The Policy Regime Framework focuses on the ideas, institutional arrangements, and interests encompassing the broad, authoritative responses to policy problems (May and Jochim, 2013). *Ideas* explain the shared understandings among different actors and decision-makers. *Institutional arrangements* are described by May and Jochim (2013) as producing “structure-induced cohesion,” which refers to the design of a particular institution and its actors. Institutional arrangements may include governmental and non-governmental entities. Lastly, *interests* include the ability of a policy regime to generate recognition or “buy-in” among the public and mobilize affected stakeholders. If the public supports the policy regime, it will often have a greater capacity to affect change; in other words, the governing capacity of a policy regime corresponds to the amount of stakeholder buy-in, or lack thereof (May and Jochim, 2013). Because of the type of data that we use in this study (policy documents^3^) and the fact that it cannot be used to infer public support, we opted to focus on the ideas and institutions of the COVID-19 policy response.

Carter and May have applied a “policy regime lens” to the COVID-19 pandemic as a theoretical exercise, an effort which we try to complement with empirical data (2020). They posit *ideas* as decision-makers discussing “flattening the curve” to reduce pressure on state healthcare systems versus the “opening [of] the economy,” which was often invoked to rebut controversial mitigation strategies, such as social distancing (Carter and May, 2020). Relevant *institutional arrangements* included (1) the apparent lack of coordination between directors of the CDC and the Food and Drug Administration during the pandemic, and (2) the ill-prepared status of the U.S. healthcare system to respond adequately to the emerging crisis (Carter and May, 2020). Last, in the case of *interests*, these included (1) the radical politicization of COVID-19 and (2) the resulting response measures, which often fell along party lines, creating a divide that failed to generate bipartisan support. Thus, federal-state relationships and governing capacity may have suffered (Carter and May, 2020).

Other studies have applied aspects of the Policy Regime Framework to analyze policy on climate change (Campbell-Lendrum et al., 2007; Wood et al., 2014), carbon sequestration (Peterson St-Laurent et al., 2017), renewable energy production (Sergent, 2014), political revolutions (Givel, 2015), U.S. National Security (May et al., 2011; Wirls, 2015) and COVID-19 (Carter and May, 2020; Cai et al., 2021).^4^

## Materials and methods

A case study design was selected since it enables analysis of current and unfolding events that cannot be manipulated (Yin, 2009). This study views policy responses to COVID-19 in the southern U.S. as a critical case, defined as a case critical to Policy Regime Theory, where a policy regime was enacted during a crisis. We expected federal, state, and local scales of the policy regime to show variation for ideas and institutions unique to their scale, offering insights that first tell us what the government has done at each scale to respond to the pandemic and how these policy responses fit together. Second, our case can lay the groundwork for comparison to responses in different regions or different countries as we begin to study the efficacy of our pandemic response–a subject that will be studied for a generation.

This case study used an exploratory sequential research design, where qualitative data is collected first, followed by quantitative analysis to further understand qualitative results. Mixed methods research is preferred when neither qualitative nor quantitative methods alone provide an adequate understanding of a complex topic (Palinkas et al., 2011). The purpose of an exploratory sequential study is that the qualitative findings (i.e., how a policy regime is enacted) can inform the quantitative method (how variation occurs at the federal, state, and local scales) (Creswell and Clark, 2017).

We collected a total of *n* = 277 policy documents, including the statements of policymakers, governmental and non-governmental organizations, and private sector actors enacting COVID-19 policy within federal, state, and local scales. One hundred ninety-six policy documents were from the federal government (71%), 31 focused on state governments (11%), and 40 focused on local governments (14%). There were also 10 documents coded as “international” (4%). The first policy document collected was published in 2016 (a document from the National Security Council on fighting pandemics) and the last collected policy document was published in July 2021 (a CDC website with updated information on how the virus spreads). We defined *policymakers* for COVID-19 as prominent governmental employees (both elected and civil service) working in an agency or organization with statutory authority or significant relationships to agencies/organizations with statutory authority. Often, policymakers work closely with the private sector (defined as a company owned by an individual or publicly traded) and with non-governmental organizations (NGOs) defined as incorporated non-profit entities.

Our sampling logic is purposeful sampling, a widely used qualitative research technique for identifying and selecting information-rich data related to a phenomenon of interest (Palinkas et al., 2015). Purposeful sampling entails selecting documents with first-hand or detailed information on the phenomenon of interest (Creswell and Clark, 2017). We determined when we had collected enough policy documents when *information saturation* was reached or until no new substantive information was entered into the dataset (Miles and Huberman, 1994). New concepts in statements stopped adding to the overall story at *n* = 248. We found that we had compiled only a limited number of policy documents from the local scale. We chose to search for and add an additional 29 local data points to the data set for a total of *n* = 277.

**Figure 1:**
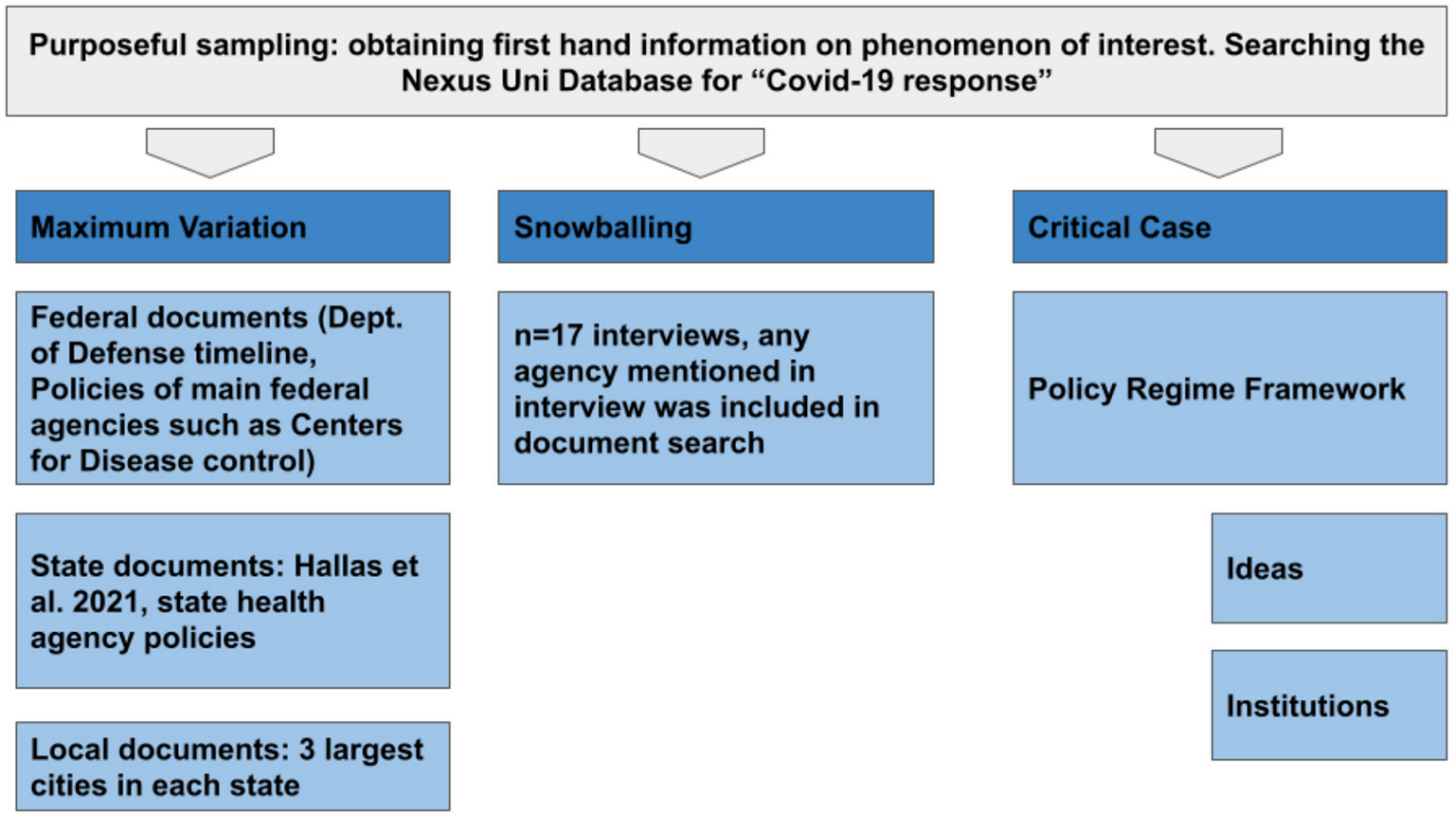
Sampling method.

**Figure 2:**
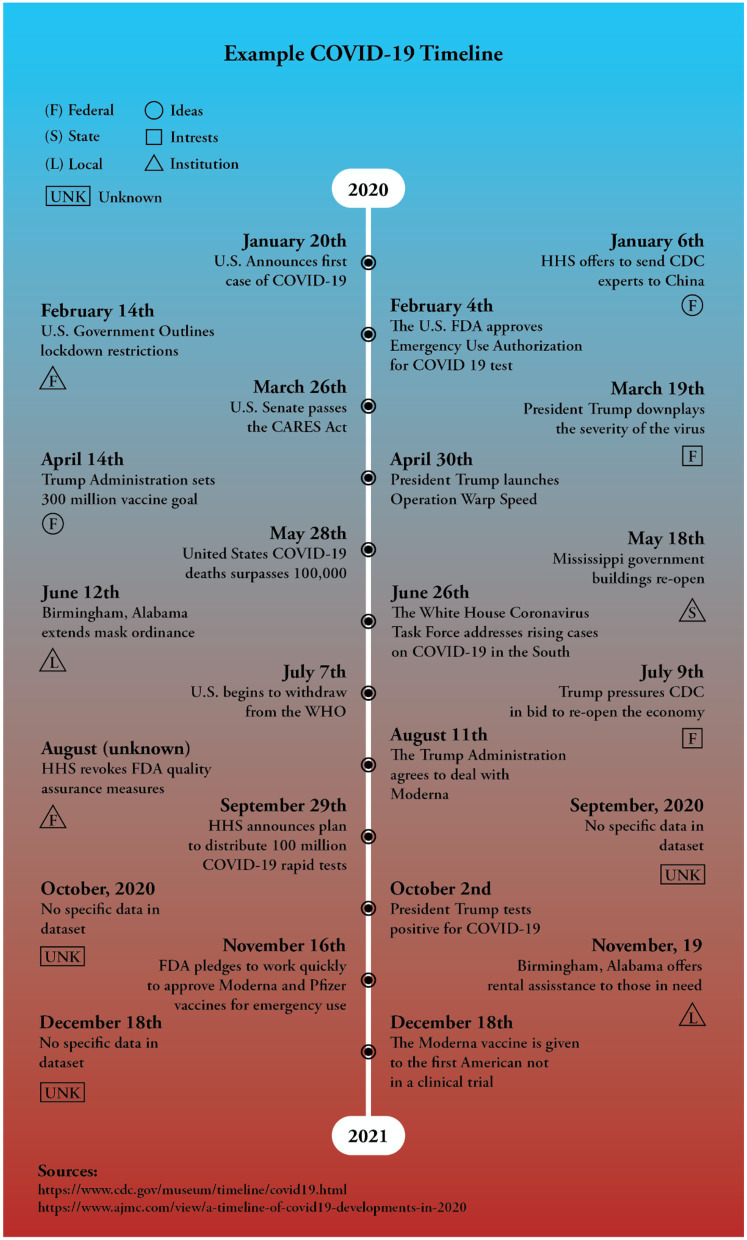
This timeline represents core events from 2020 to 2021. The central events along the timeline were sourced from the CDC and the American Journal of Managed Care (AJMC Staff, 2021; Centers for Disease Control Prevention, 2022b). The exterior events are examples from our data, representing how *ideas, institutions*, and *interests* can provide context for government responses to the COVID-19 pandemic.

To build our dataset of policy documents, our purposeful sampling strategy combined three specific, purposeful sampling strategies: maximum variation, snowballing, and critical case strategies (Palinkas et al., 2015) (see Figure 1). Beginning with maximum variation, we relied on this strategy to seek out important shared patterns that cut across policy documents that derive their significance from their heterogeneity. In our research, this heterogeneity involved clustering policy documents into the federal, state, and local scales of government. It required exploratory research on COVID-19 policy responses to facilitate our understanding that policy responses are likely varying at these three scales. For federal policy responses, we used the Department of Defense policy response timeline, which comprehensively lists all federal responses to COVID-19 (U.S. Department of Defense, 2022). For state-level responses, we used the working paper published by Hallas et al. (2021) outlining the U.S. state policy responses to COVID-19. For local responses, we performed targeted searches of local government websites for the three largest metropolitan areas in each state (Alabama: Huntsville, Birmingham, Montgomery; Louisiana: New Orleans, Baton Rouge, Shreveport; Mississippi: Jackson, Gulfport, Southhaven) (see Figure 2). These searches took place between March and July of 2021. A difficulty in this approach is that as government officials and administrations change, so do the policy documents listed in timelines and on government websites. Many government websites only show the most recent recommendations without a way to access prior information. This was mitigated by copying the text of policy documents into our data set and categorizing it using dates (for both publication and date of access) and agency names.

Once this preliminary understanding was obtained, we then explicitly sought out policy documents from specific federal agencies (e.g., CDC, National Institutes of Health, and Federal Emergency Management Agency); from state agencies (e.g., Louisiana Health Department); and from local governments (e.g., City of Birmingham, Alabama). To ensure that we were collecting documents in a valid way, we triangulated our compilation using snowballing. Snowballing was used during the creation of this dataset by performing key informant interviews of 17 respondents. The human subjects interview data collected from this process was not used in this study, but rather the names of the agencies that these respondents work for and the snowballed organizations that they named as important epicenters of COVID-19 policy responses were collected. We asked key informants, “who knows about the COVID-19 policy responses in Alabama, Mississippi, and/or Louisiana?” and their answers determined where we would sample relevant policy documents. The 17 key informant interviews were conducted remotely using ZOOM video conferencing technology between December 2020 and June 2021. Respondents were identified based upon representation of the largest population centers within each state. They included local government employees and elected officials, state and regional public health officials, and employees of local and regional media outlets, universities, NGOs, and local and regional business associations. All respondents verified that they had been responsible for communicating COVID-19 information to the public in a policy-making organization or role.

In addition to using key informants to help us sample and select policy documents, we used two comprehensive timelines to cross-check human subjects' data and help us determine when information saturation had been reached at the federal, state, and local scale. These timelines included (1) that of the Department of Defense, which comprehensively lays out all federal responses to COVID-19 (“Coronavirus Timeline,” n.d.) and (2) that of Just Security, a think tank based at the Reiss Center on Law and Security at the New York University School of Law (Goodman and Schulkin, 2020).

To narrow down thousands of policy responses and potential documents, we used a third type of purposeful sampling strategy called critical case thinking. Critical case strategies for sampling permit logical generation of data and analysis, assuming that our findings of the policy regime in Alabama, Mississippi, and Louisiana may also be relevant to other cases (Palinkas et al., 2015). Using these methods, we could then frame the COVID-19 policy responses within federal, state, and local contexts with the Policy Regime Framework and its components (e.g., ideas and institutions). In order to be included in our dataset, policy documents needed to speak to at least one of these concepts.

Twenty-two specific search terms were entered into the online search engine Google and the Auburn University Library Nexis Uni Database using the terms “COVID-19 response” with the name of the organization, agency, or jurisdiction. Search terms included are listed in Appendix A. There were criteria for the types of policy documents utilized in this search. Policy document selection criteria included documents, websites, social media posts, and videos that tell what agencies within the government are doing to respond to COVID-19.

We coded the documents using the Policy Regime Framework theoretical constructs. Coding took place in two cycles. In cycle one, we looked for “lenses,” or the components of the Policy Regime Theory, specifically ideas and institutions (Strauss and Corbin, 1997). The codebook, which contains the main themes from the Policy Regime Framework is located in Table 1. In brief, policy documents received the code for “ideas” if a document mentioned science, research, or technical information underlying response to COVID-19. Policy documents received the code for “institutions” if they were related to an agency's activities, procedures, or if they contained information about an agency's work across scales (federal, state, and local). In some places, “interests” were revealed by way of policy-maker actions (e.g., cutting federal pandemic response programs before the pandemic shows some voters/elected leaders' interests in minimizing the role of the federal government). Where possible, we note in our analysis where these interests break through. To determine where they break through, we use the “3-i Framework” criteria for defining interests, which asks: who wins/loses, and by how much do some win and some lose (Gaynor, 2020).

**Table 1:** Codebook for the Policy Regime Framework.

**Concept**	**Criteria to receive that code**
Ideas	Code for IDEAS if a document mentions the science, research, or technical rationale behind an agency's response to COVID-19 IDEAS example: any scientific data to inform lockdowns, quarantines, hand washing, opening/closing schools, opening/closing economies
Institutions	Code for INSTITUTIONS if an agency's activities, procedures, responses, collaborations of the agency is mentioned (anything the agency itself is actually doing to implement policy). Sometimes the document itself may be the agency's action if giving information is their role (CDC) Code for INSTITUTIONS if an agency's activities working across scales are mentioned (federal-state collaborations, state-local collaborations) INSTITUTIONS example: The Food and Drug Administration expediting the vaccine approval, FEMA opening up vaccine centers, the university opening up dorms to quarantine

This research was a multi-coder effort with multiple coders assessing inter-coder reliability. Seven total coders assigned codes between March and June 2021, and three of the seven coders selected approximately 25% of codes to check the work and ensure agreement between the previous coder.

After the first sorting of theoretical concepts, a second round of coding took place. Respondents' own words were used to further sort the theoretical concepts into smaller components, preserving the participant's perspective and helping to understand how ideas and institutions were defined at the three scales of government (Saldaña, 2021). Phase two required that we refine the initial codes to what Saldaña refers to as “consolidated meaning,” where you group similar codes within an overarching category.

In order to further narrow down the large quantity of information generated from our policy document dataset, we used an automated text analysis method to efficiently extract common themes (topics) from the reviewed literature. We used the R package *stm* (Structural Topic Models; Roberts et al., 2019) to identify co-occurring content and prevalence of these topics within these framework levels. The structural topic model allows us to identify topics using document-level metadata (Roberts et al., 2019). To incorporate document-level metadata, data for the first round of coding (for the Policy Regime Framework levels: *ideas and institutions)* were used. We then identified whether the policy document was sourced from federal, state, or local sources. We used the most common two-word phrases at each scale of government for ideas and institutions to form our thematic (topic) model. Two-word phrases allowed us to better make sense of the data, as single-word outputs were less relevant.

## Results

### General results

The main actors at the federal level include former President Trump, the CDC, the Federal Emergency Management Agency (FEMA)^5^, Congress, and individuals who represent those organizations. At the state level, leading actors included governors, legislatures, universities^6^, state hospital systems, and public health agencies. Lastly, at the local level, actors included city governments, officials, hospitals, local news stations, and newspapers.

There were two distinct periods in the U.S. during the COVID-19 pandemic. The first period focused on specific safety actions prior to the rollout of the vaccine. These actions included (but were not limited to): wearing a mask, social distancing, closing schools and businesses, restricting travel, quarantining, stay-at-home orders, testing, and increasing ventilation (Mississippi State University, 2020; Centers for Disease Control Prevention, 2020c; Alabama Department of Public Health, 2009, 2021; American Red Cross, 2021; Occupational Safety and Health Administration, 2021; City of Birmingham, 2021b; World Health Organization, 2021b). We examined which levels of government used these safety actions in their messaging. The second period of messaging took place after vaccines had become available to the public in mid-December 2020 (Centers for Disease Control Prevention, 2020a). After the administration of vaccines began, messaging shifted to promoting vaccine trust and confidence as well as increasing overall vaccine acceptance (Centers for Disease Control Prevention, 2020b).

### Ideas

#### Federal messaging on ideas: Declaring an emergency, suggesting safety responses, providing supplies

Of the 277 policy documents examined, 170 mentioned *ideas* (i.e., science, research, or technical rationale behind an agency's response to COVID-19), making this the primary messaging topic. Because the messaging of federal agencies focused mostly on communicating ideas^7^, it makes sense to ask, “what were the leading perceptions of the core ideas behind policies in regard to the COVID-19 regime?” (May and Jochim, 2013). Table 2 below provides an overview of these ideas.

**Table 2:** A list of qualitative codes and examples for *ideas* at the federal, state, and local levels.

**Ideas**
**Level**	**Qualitative code examples**	**Examples**
Federal	Pandemic preparedness; COVID-19 spread; COVID-19 origins; emergency declarations; asymptomatic transmission; travel bans; safety measures; experimental treatments; testing; social distancing; stay-at-home orders; vaccine goals; business guidance; comorbidities; minority community susceptibility; mask wearing; new agency guidelines; vaccine progress	1). “Based on current information, the risk from [COVID-19] to the American public is currently deemed to be low. Nevertheless, CDC is taking proactive preparedness precautions. Entry screening is part of a layered approach used with other public health measures already in place to detect arriving travelers who are sick to slow and reduce the spread of any disease into the U.S” (Centers for Disease Control Prevention, 2022a). 2). “A layered strategy combines multiple prevention strategies such as consistent and correct use of masks, ventilation, physical distancing, cleaning and disinfection, and hand hygiene” (Centers for Disease Control Prevention, 2021).
State	State university data; state agency data; “flattening the curve”; shelter-in-place; COVID-19 health risks; COVID-19 trends; COVID-19 statistics; PPE effectiveness; monitoring programs; CDC and state health department guidelines; data guiding decision-making	1). “Data collected through the [Louisiana State University] Daily Symptom Checker, the Louisiana State University Emergency Operations Center, and COVID-19 testing centers will help drive the university's decisions about mitigation strategies and operations” (Louisiana State University, 2021). 2). “Based on what's been seen in […] Seattle and Wuhan, China, only a portion of people who pick up the coronavirus will have serious symptoms. Only a portion of those who are hospitalized will need intensive care, and a portion of those will need ventilators” (Vaughan, 2020).
Local	Self-screening; self-reporting; proper PPE usage; types of PPE available; following state and federal guidelines; changes to daily routines; COVID-19 incubation period; equal access to COVID-19 data; restaurant precautions; restaurant capacity; COVID-19 incidence rate	1). “It is critically important that you and your family members understand this virus moves quickly and is potentially deadly, especially to the elderly, people with diabetes or cancer, and those who have weakened immune systems. Just because you feel healthy doesn't mean you're not a carrier of this virus […]” (Bryan, 2020). 2). “It's an effort [the Mayor] says right now is necessary. [At] the rate that we're going, the cases that we're seeing may result in the loss of thousands of lives. We're trying to prevent that” (Bowerman, 2020).

At the federal level, the most commonly used messaging topics (two-word phrases isolated using automated text analysis and our own multi-coder effort) were *public health, social distancing, infectious diseases, safety actions*, and *medical supplies*. This suggests that federal agencies were focused on messaging that (1) communicated to the American public that a public health emergency in the form of a major disaster was unfolding, and (2) promoted safety actions such as social distancing and provisioning of supplies in an emergency capacity. The federal government issued 57 concurrent Major Disaster Declarations (in all 50 states, 5 territories, indigenous tribes, and Washington, D.C.) in 2020 (Gaynor, 2020)^8^. The following quote from *The New York Times* is attributed to senior White House officials and provides an example of recommendations for what actions to take in this Major Disaster Declaration (*social distancing* and *safety actions*):

By the third week in February, the administration's top public health experts concluded they should recommend to [Former President] Trump a new approach that would include warning the American people of the risks and urging steps like social distancing and staying home from work (Lipton et al., 2020).

Other *safety actions* prioritized by the federal government included social distancing, testing for the illness, new and increased cleaning procedures, and travel restrictions. Leadership on designing safety actions came from the CDC and the Department of Health and Human Services more broadly. Safety actions were then further spread to the general public more broadly by national and local news sources.

#### Subnational state messaging on ideas: Implementing safety responses by partnering with major institutions and the private sector

It was at the state level that federal-level *ideas* became concrete policy responses. The most common two-word phrases for ideas in subnational (state) governmental responses included: *public health, social distancing, contact tracing, health care*, and *disease control*. State governments focused on making policies, laws, and regulations requiring specific safety actions (based on federal ideas emanating from the CDC). For example, the governor of Alabama, Kay Ivey, issued a mask mandate on July 16th, 2020, that ordered masks be worn in public indoor spaces, on public transportation, in gatherings of 10 or more people, and in outdoor public spaces (Lardieri, 2020). Mississippi and Louisiana also issued state-wide mask mandates in the summer of 2020 (Louisiana Office of the Governor, 2020; Exec, 2020). These mandates were based on CDC science and recommendations that individuals should wear masks to help prevent the spread of the virus (Centers for Disease Control Prevention, 2020c).

As a public safety action, contact tracing was often employed by the agencies responsible for disease mitigation and public health, such as state health departments (Louisiana Department of Public Health, 2021). Compared to more uniform mask mandates, contact tracing has taken on several forms and has been used for decades by state and local officials to stop the spread of infectious diseases. This method identifies people who may have been exposed to a pathogen and alerts them to quarantine and to monitor their health for signs and symptoms (Centers for Disease Control Prevention, 2020b). Alabama, Louisiana, and Mississippi all conducted contact tracing differently, although the *ideas* and much of the financing came from the federal government (CARES Act, 2020). These policy responses ranged from partnering with large state institutions, such as major university systems, to performing the task within the state government agencies themselves. For example, the state of Alabama has partnered with the University of Alabama at Birmingham to conduct its contact tracing efforts (Windsor, 2020). On the other hand, Louisiana has outsourced its contact tracing to the private sector, opting to hire contractors (Myers and Sledge, 2020). Mississippi used its own state public health governmental agency, the Mississippi State Department of Public Health (Mississippi State Department of Health, 2021).

#### Local messaging on ideas: Face coverings

The most common two-word phrases used by local government entities were *face coverings, neck gaiters, face shields, positive cases, medical guidance*, and *number active* (the software used for analyzing the data removed words like “of”). Local government focused on ensuring that citizens understood how to engage in safety actions in relatable and practical examples. Local governments focused on providing *ideas* for how individuals without access to surgical masks can still comply with state mask mandates. The following quote from a New Orleans government official quotes CDC guidance in a general way, making it more easily digestible:

Current CDC data suggests that a cloth face covering may protect the wearer and prevent the spread of the virus to others. Visit CDC's [“do it yourself”] Cloth Face Coverings [website] to see CDC guidelines on the use of face coverings (Mississippi State Department of Health, 2021).

Even when state mandates for face coverings ended, some private sector businesses kept their mask mandates in place. For example, business advocacy groups like the Alabama Retail Organization, a group that is similar to a statewide chamber of commerce, provided scientific information to enable business owners to decide whether to keep a face-covering mandate in place in their stores (Alabama Retail Association, 2021). They also provided infographics and resources for business owners, such as signs to hang in their stores.

### Institutions

#### Federal government

Two hundred thirty-three out of the 277 total data points included mention of *institutions* (i.e., agency activities, procedures, responses, anything an agency did to implement policy). Federal level messaging on *institutions* focused on *public health*, the *White House*, the *Task Force*, the *private sector*, and *national security*. Table 3 above provides an overview of institutions with examples.

**Table 3:** A list of qualitative codes and examples for *institutions* at the federal, state, and local levels.

**Institutions**
**Level**	**Qualitative code examples**	**Examples**
Federal	Budget cuts; agenda setting; agency assessments; national programs; public health declarations; task force formation; press releases; testing development; travel bans; disease surveillance; interagency cooperation; private sector involvement; economic relief; congressional legislation; executive orders; PPE distribution; immigration control; vaccine development; federal guidance; emergency use authorizations; congressional testimony	1). “[Vice President Michael Pence] also announced that the Office of Management and Budget would issue guidance directing agencies across the federal government “to review internal travel policies and to adhere to State Department advisories with regard to international travel” (Chalfant, 2020). 2). “A year later, [Federal Emergency Management Agency] continues working with state, tribal, and territorial authorities to bring this pandemic to an end. One strategy is speeding up vaccinations by supporting states as they open community vaccination centers across the country” (Federal Emergency Management Agency, 2021).
State	State university responses; stay-at-home orders; state checkpoints; state executive orders; PPE distribution; PPE manufacturing; financial assistance; unemployment benefits; state prison conditions; COVID-19 testing; business restrictions; church restrictions; hospitalization; nursing homes; healthcare capacity; cooperation (multi-state, federal-state, inter-agency); timelines for “reopening the economy”	1). “At this point in the pandemic, our three best tools for slowing the spread of COVID-19 and keeping our hospitals operational are vaccinations, masks, and distance said [Governor John Bel Edwards]” (Louisiana Office of the Governor, 2020). 2). “The nation‘s governors are in talks about creating a multi-state consortium to oversee the purchase and distribution of medical supplies across the country—a direct response to the White House's hands-off approach to the issue” (Gronewold, 2020).
Local	Curfews and exemptions; shelter-in-place orders; social distancing; community guidelines; limited public/private gatherings; daily screenings; business protocols; university operations; public school operations; “personal responsibility”; drive-in testing; PPE orders and distribution; alternative modes of education; health care capacity; local press releases; state(s) of emergency; food assistance; essential vs. non-essential businesses; town halls; small business loans	1). “[New Orleans] Adjusts Gathering Size and Capacity Limits Under Modified Phase III Guidelines: Effective April 2, all indoor public and private gatherings shall be limited to 150 individuals […] Outdoor Recreation Spaces and Sports Complexes will be allowed to open at up to 50% of standing capacity” (City of New Orleans, 2020). 2). “Mississippi Gov. Tate Reeves, a Republican, has designated churches as essential, allowing them to operate as long as they follow state and federal health guidelines. The city of Greenville, however, has barred churches from holding either in-person or drive-in services as long as the governor's shelter-in-place order remains in effect.” (Williams, 2020).

The most important institution was the U.S. White House, with influence in American public life preceding the pandemic. The Trump administration disbanded the institution responsible for pandemic response: the National Security Council's Global Health Security and Biodefense Unit^9^. American lawmakers expressed concern to President Trump, such as in a letter from Senator Sherrod Brown in 2018 where he cited the importance of this unit to address international health crises such as the Ebola virus (Brown, 2018). Senator Brown's letter also criticized proposed budget cuts, arguing that these would leave Americans vulnerable to the “next, inevitable outbreak.” Other Congressional Lawmakers expressed similar concerns worrying that “fragmented organization of global health security responsibilities throughout the federal government” may characterize a future pandemic (Bera and Connolly, 2018). At the same time, Rear Admiral Timothy Ziemer, the only senior national security official focused on pandemic preparedness, was removed from his post, and no replacement was assigned (Reuters, 2020). The Trump administration also proposed cuts^10^ to the CDC's Prevention and Public Health Fund, a fund that partially supports immunization access and infrastructure (PBS NewsHour, 2018). These examples show how the President's policy agenda-setting, staffing decisions, and priority-setting directly contribute to disaster preparedness and response. Although our manuscript does not focus on interests, these policy-making events depict interests of the U.S. president's political party, the Republican Party, a party with a platform typically focused on reducing the size and scope of the federal government. Likewise, research has shown that public attitudes of Republican voters that trust the federal government to manage the pandemic have a 25-point gap compared to Democratic voters, a significant difference revealed in the actions of their elected leaders (Hamilton and Safford, 2020). Thus, Trump voters may perceive a win in these cuts, but those who opposed Trump and his party may see a loss of essential services during an emergency.

#### Pre-vaccine pandemic

One of the White House's initial responses was to create the President's Coronavirus Task Force, which was designed to bring together federal actors and pandemic experts to inform the White House's response to the pandemic (this is expanded upon in the following section).

Speaking to the public and presenting information is also one of the key institutional actions of the presidency, especially during national emergencies such as a pandemic (Bucy, 2003). President Trump used his pulpit to speak to the American public to assuage public fear toward the virus and reassure the public that the federal response was highly effective. His language would often downplay the severity of the pandemic. Below is an example from the data of the language President Trump employed:

You may ask about the coronavirus, which is very well-under control in our country. We have very few people with it, and the people that have it are … getting better. They're all getting better. … As far as what we're doing with the new virus, I think that we're doing a great job (Blake and Rieger, 2020).

The President used his office to curate federal-level coronavirus communication and messaging. The White House sparred over language use with the CDC on numerous occasions, including on CDC guidelines for religious services that initially recommended less singing during services and that members not share drinking cups (Sun and Dawsey, 2020). After pushback from the White House, the guidelines were changed to clarify First Amendment protections and to have no mention of choirs or singing. CDC officials were asked to clear formal documents and guidelines with the White House before anything was released.

Two major policy responses that the White House enacted included travel bans and the initiation of Operation Warp Speed. The first ban was for travel to and from China in early February 2020, and a second quickly followed, extending the ban to Iran (Facher, 2020). In March, another ban was announced for 26 European states (BBC News, 2020). These travel bans were initiated to stop new coronavirus cases from entering the U.S. However, they were implemented after the first cases of the virus were already reported within the U.S. in January 2020. The White House initiated Operation Warp Speed on May 15th. It aimed to bring together and organize government agencies, the military, and pharmaceutical companies to accelerate the development of a COVID-19 vaccine (Jacobs and Armstrong, 2020).

These data demonstrate various ways the President used his office to address COVID-19. Concrete actions such as designating a Task Force and launching Operation Warp Speed are key ways the White House influenced federal response to the pandemic. These findings are coupled with overarching policy preferences for smaller government, proposed budget cuts, and personnel arrangements that set the stage for White House operations and efficacy. These elements, mixed with the President's ability to craft public messaging, demonstrate the importance that the Presidency has in pandemic response, even before a pandemic has occurred.

#### Task force

On January 29, 2020, President Trump created the President's Coronavirus Task Force to manage, mitigate, and oversee federal response to the virus (The White House, 2020a). It was staffed by a variety of government professionals and scientists from the federal government, including Dr. Deborah L. Birx, then the State Department's AIDS director; Alex Azar, then United States Secretary of Health and Human Services; and Dr. Anthony Fauci, who since 1984 has served in a leadership role in the National Institutes of Health. Additional members are listed in Appendix B. The focus of the Task Force was on border control as a means to stop the spread of COVID-19.

The Task Force was the main federal institution briefing state leaders (such as the National Governors Association) on the most recent science and responses required of the federal and state governments (Department of Health Human Services, 2020). As soon as February 21, 2020, the Task Force began to discuss that the federal response should consider shifting solely from international border control and containment of the virus through various travel bans to “mitigation,” meaning the implementation of social distancing mandates among the U.S. public (Lipton et al., 2020). On February 26, a meeting that would recommend social distancing to President Trump was canceled, and Vice President Pence replaced Alex Azar as head of the Task Force. Azar's Task Force had previously received criticism from the White House for advocating public health measures that the White House felt were too extreme (Diamond, 2020). The next day, February 27, Vice President Pence added Larry Kudlow, an economic advisor to President Trump, and Treasury Secretary Stephen Mnuchin to the Task Force to ensure that the economy remained a key consideration (Collins and Vazquez, 2020). On March 2, Vice President Pence officially recognized mitigation as the Task Force and U.S. Government's new goal. Shortly after, the Task Force began planning mitigation strategies for hard-hit communities (including the U.S. Southeast) across the U.S., including aims to expand testing and sending PPE to those in need (Schwellenbach, 2020; The White House, 2020b). In early May, Vice President Pence suggested that the Task Force would finish its work by the end of the month (Weiland et al., 2020). However, it was quickly decided that the Task Force would instead shift focus from mitigation to “re-opening” the country and the economy (Cillizza, 2020).

Through the summer and fall of 2020, the Task Force would continue to advise federal response to the pandemic. This occasionally resulted in criticism of inconsistent messaging from the White House, which would recommend actions that directly conflicted with guidelines laid out by the CDC, the agency that would traditionally lead a response to a pandemic in the U.S. This criticism came from CDC officials, aides who left the White House, state governors, and unnamed individuals who were purportedly close to Task Force discussions. For example, in March 2020, the Task Force and the CDC simultaneously issued different numbers and size recommendations for social gatherings (Mazzetti et al., 2020). In October 2020, the Task Force refused to legitimize a CDC mandate to require employees and passengers to wear masks on all public and commercial transportation (Kaplan, 2020).

The creation of the Task Force was a central institution-oriented action taken by the federal government to address the COVID-19 pandemic. Its frequent appearance in the data demonstrates the Task Force's importance. The narrative laid out by the data shows how the Task Force was largely involved in every aspect of the federal response, at times openly contradicting scientific institutions such as the CDC. President Trump's Task Force openly contesting the CDC, an agency which Republican voters do not place trust in Hamilton and Safford (2020), again reflects the political interests breaking through policy-making. Voters who perceive the federal government as untrustworthy perceive the President contesting leadership, and with that, a political victory. This dynamic creates losses for citizenry seeking a clear and transparent message from their elected leaders.

#### Private sector as a federal partner for implementation

Following the longest recorded economic expansion in U.S. History (2009–2019) and the subsequent outbreak of COVID-19 in 2020, the U.S. saw the most significant drop in Gross Domestic Product (GDP) since the measure was created (Bauer et al., 2020). Between February and April 2020, the U.S. lost 22 million jobs, resulting in an economic crisis that disproportionately impacted women, minority workers, lower-wage earners, and less educated people (Bauer et al., 2020; Stevenson, 2020). The private sector was closely tied to federal institutions because the U.S. government relied heavily on the support of the private sector to meet objectives for COVID-19 testing, enact PPE production and distribution, and conduct vaccine research and production. To ensure effective collaboration between the federal government, the FEMA Supply Chain Task Force was created, headed by Jared Kushner, President Trump's son-in-law (Cancryn and Diamond, 2020). This institution enacted airlifting emergency medical supplies to the U.S as a part of Project Airbridge, crowdsourcing PPE donations, establishing drive-through testing sites, and quickly devising hospital plans to maximize ventilator usage. This Task Force received criticism because its authority overlapped with existing disaster response procedures and personnel within FEMA and the Department of Health and Human Services (Cancryn and Diamond, 2020). This approach complicated federal agencies' abilities to respond to COVID-19 by decentralizing projects and creating jurisdictional confusion (Confessore et al., 2020).

#### State government institutions

The most common two-word phrases about institutions at the state level focused on *face coverings, social distancing, campus community, staff* and *students*, and *exposure notification*. Because our sampling purposely focused on universities, it is not surprising that many of our data points mentioned *campus community, staff, students*, etc.

The most important state-level institution was found in the executive branch, specifically in state governors and public health agencies. State public health agencies issued regulations in the form of Emergency Orders, which are executive orders or regulations that allow lawmakers flexibility and rapid action by bypassing the legislatures. The priorities of Emergency Orders focused on the two-word phrases from our data (e.g., face coverings, social distancing, exposure notifications). In general, Alabama, Mississippi, and Louisiana's Emergency Orders were administered in two phases: year one of the pandemic, between March of 2020-March 2021, when the public was asked to remain at home (except for “essential workers” such as healthcare workers), and from April 2021 onward, where the public was advised to stay home if they would like to diminish risk– the former constituting a period of higher risk and uncertainty. For example, the Alabama Department of Public Health issued its first Emergency Order on March 6, 2020, alerting healthcare workers and the public that a novel disease outbreak was underway and that depending on the severity of respiratory symptoms being experienced, officials in state or local government should be notified within several hours to help monitor case counts in the state (Alabama Notifiable Diseases/Conditions, 2020). Year one of the pandemic in Alabama was characterized by Emergency Orders known as *Safer at Home*, asking residents to stay home unless their job required them to be in public and limiting gatherings in places like religious buildings and gymnasiums. Year two Emergency Orders were titled *Safer Apart* and were less strict than the previous year.

The second type of important institutions at the state level included public, state government-administered university systems, and state agencies responsible for public health policies. American universities are important places to study the pandemic policy responses as they house approximately 20 million students, many of whom are in close quarters in classrooms and shared housing (Smalley, 2021). Our data focuses on public flagship universities, which in the U.S. context are known as leading national or regional universities dating back to the founding of public universities in the U.S. in the mid-1800's (Douglass, 2016). Due to our purposeful sampling strategy, our data focuses on policy responses related to university policy responses, leaving school-age children outside of the scope of this paper, despite the issue's indisputable public importance.

Public universities in the U.S. had policy responses set within two discrete periods: before and after the vaccines. Prior to the vaccine, universities issued guidance based on the *ideas* or the science emanating from the CDC. University guidance often accommodated different instructional modalities (such as moving classes online or a hybrid of online and in-person), safety actions (such as requiring mask-wearing and social distancing), and technologies such as smartphone apps to enable students to self-screen for symptoms before coming to class.

Following the rollout of the vaccine, universities attempted to persuade their students and nearby communities to get the vaccine. This was important because of the remarkably low vaccination rates in the Southeastern U.S. Alabama, Mississippi, and Louisiana were in the bottom seven U.S. States for percent of the population fully vaccinated in late 2021 (The New York Times, 2021). One example of a persuasion campaign is Auburn University in Alabama which partnered with famous alumni and basketball athlete Charles Barkley to communicate with students about the safety and efficacy of vaccines (Auburn University, 2021).

#### Local government: Specific actions to keep the public safe

The most common two-word phrases about institutions at the local government level were *sports complexes, recreation spaces, private gatherings, standing capacity*, and *health department*. This furthers the pattern wherein state and local governments adopted their *ideas* from federal actors, issued Emergency Orders from state executive branches of government, and implemented and enforced them at the local scale. One such way was limiting the number of people allowed in indoor spaces at any given time. These limitations were often made by local governmental actors complying with state-level Emergency Orders. An example can be found in the statement below from the New Orleans local government:

All indoor public and private gatherings shall be limited to 150 individuals and outdoor public and private gatherings shall be limited to 250 individuals. Also, Outdoor Recreation Spaces and Sports Complexes will be allowed to open at up to 50% of standing capacity (City of New Orleans, 2020).

Local governments were focused on compliance with specific safety actions and mandates. Throughout 2020, local governments (city and county governments) in the southeastern U.S. implemented many different safety precautions and mandates. For example, Jackson County, Mississippi, asked visitors not to enter county buildings, and employees were required to participate in daily screening (Jackson County Mississippi, 2021). In Jefferson County, Alabama (city of Birmingham), the Department of Health began providing COVID-19 testing for children (City of Birmingham, 2021a). Furthermore, Auburn, Alabama, closed the public library in March 2020 and shifted its purpose to being a COVID-19 resource center for residents (Dorton, 2020).

Despite mask mandates being issued by state governments, enforcement became the responsibility of local governments. Similar to states partnering with large institutions like universities, local enforcement often happens with large institutions and the private sector. For example, the largest and most well-known retail store in America, Walmart, issued its own mask mandate in its stores on July 20th, 2020 (Smith and de la Rosa, 2020), which occurred earlier than the state mandate for face coverings in Mississippi (August 4th, 2020). Ninety-five percentage of Americans shop at Walmart and it is also the primary retailer for rural and low-income Americans, highlighting the importance of Wal-Mart's actions for local government initiatives (Emory, 2017; Gustafson, 2017). Private sector businesses operating at Walmart's scale requiring face covers may even act as the de-facto enforcement of state and local orders.

#### Ideas and institutions at each level of government: Similarities and differences

Ideas in the Policy Regime Framework are where the science, research, or technical rationale behind an agency's response to COVID-19 are found. For the federal government, the focus was on the formal declaration of a national emergency, suggesting safety responses through input from federal agencies, and providing medical supplies to hospitals, state governments (e.g., state health agencies), and other healthcare facilities (e.g., nursing homes). It was at the state level where the federal-level safety suggestions became concrete policy responses for local governments to implement. Lastly, it was the local level of government where officials ensured that citizens understood how to best engage in safety actions (e.g., masks and social distancing).

Institutions in the Policy Regime Framework are most commonly associated with the agency activities, procedures, responses, or anything an agency did to implement policy. At the federal level, messaging around the concept of institutions focused on the White House in two main ways: (1) its creation of the White House Coronavirus Task Force that brought together federal officials, public health officials, and pandemic experts and (2) the Trump administration's travel bans and vaccine development. At the state level, messaging around the concept of institutions focused on two main points: (1) governors and other state-level agencies issuing Executive Orders and other concrete policies and (2) state university systems implementing safety measures, altering teaching modalities (e.g., in-person vs. online), and rolling out vaccines for the student populations and surrounding communities. Lastly, local governments messaged around the idea of institutions in one main way, focusing on implementing public health measures to protect local communities (e.g., limiting the number of people in public areas, closing government buildings for in-person services, closing recreational spaces, and implementing mask mandates for local businesses).

## Discussion

At its core, the analysis of policy regimes asks, “How do significant shifts in public policy occur?” Wilson (2000) suggests that significant policy regime changes operate based on paradigm shifts, where catastrophic events, demographic challenges, economic crises, and other policy problems act as flash-points for revolutions in policymaking. The beginning of the COVID-19 pandemic served as a flash-point for policymakers, as they were tasked with responding quickly to a novel and deadly virus. This is important to note because in a scenario like a pandemic, situations on the ground change rapidly and governmental turnover results in policy documents and policy recommendations being erased, modified, or obscured. Our research contributes to this thinking by theorizing how significant policy regimes are enacted. In our research, ideas emanated from the federal government (The CDC and White House), which, at times, could not agree on the ideas to inspire policy. Ideas were used by executive governments in the states to enact Emergency Orders, which initially led to strict orders (stay-at-home orders and restricting gatherings) that were eased over time. At the local level, the responsibilities for enforcement of state orders were borne by local businesses and government. Additionally, as administrations changed at every level of government following the 2020 election and the pandemic continued, entire websites and policy documents were deleted or taken offline. This further exacerbated the difficulties faced by decision-makers as they continued to deal with vaccine hesitancy and decisions related to public health, such as easing social restrictions. Further research is needed to determine whether, in states like Alabama, Mississippi, and Louisiana, with often vulnerable, marginalized, and low-income populations, these communities were best served by this policy regime response to COVID-19. For instance, with White House officials battling with experts in the CDC for weeks over reopening guidelines (Sun and Dawsey, 2020), were these communities in the Southeast caught in the middle, bearing the impacts of these disagreements? An initial look at this question portrays a regional impact that is disproportionate to the other regions in the country. The U.S. Southeast has the lowest vaccination rates in the country with Alabama, Louisiana, and Mississippi in the bottom four in terms of percent fully vaccinated. Additionally, Mississippi has the highest death rate from COVID-19 in the country with Alabama and Louisiana also in the top 10. We cannot say for certain if these numbers are a direct result of governmental messaging around the seriousness of the virus or the importance of getting vaccinated, they do indicate that more research is needed to determine exactly what impact messaging had on these populations.

It is also suggested by May and Jochim (2013) that as new policy regimes arise, policy effectiveness hinges on the feedback processes that influence those governing arrangements. We corroborate their argument with several of our findings from the federal-level institutions data. First, with Former President Trump frequently downplaying the severity of the COVID-19 pandemic, and with his administration cutting key pandemic preparedness bodies within the government, it is possible that these actions led to feedback all over the country. In Alabama, for example, non-Hispanic Black Americans without postsecondary education perceived themselves to be at less risk from COVID-19 compared to other groups (Scarinci et al., 2021). Is it possible that the White House downplaying the virus informed these perceptions? Further research can determine whether the specific communication strategies and content of messages led to behavioral changes in the public that put already at-risk communities at greater risk.

The need for time-sensitive, rapid research has been identified by other authors, even in the context of COVID-19. Rahman et al. (2021) emphasize that in order for decision makers to make informed choices, researchers must employ innovative, methodologically-sound strategies to quickly communicate accurate information. Furthermore, developing these research skills helps to prepare the scientific community, as well as our governing bodies, to respond to crises like the COVID-19 pandemic in the future.

There are many difficulties associated with designing, implementing, and disseminating RAPID studies during a crisis. These challenges include time pressures, changing governments, limited resources, and the ability for decision-makers on the ground to receive and digest the research findings. As the pandemic went on, new variants of the SARS CoV2 virus emerged, causing decision-makers to change their approaches to mitigation and responses to governmental directives. As this happened, it was difficult to stay up-to-date on governmental policy responses because policy documents were removed, policies changed, and information was difficult to find. For example, the Executive Order signed by the mayor of Carencro, LA, a suburb of Lafayette, LA, that laid out policy guidance for the public has been removed from the government's website and is no longer accessible. Another example of data becoming increasingly difficult to access is on the New Orleans, LA Chamber of Commerce website. The COVID-19 Resources section of its website is still active but has removed links that were originally under “COVID-19 Website Resources.” There were also a number of policy documents that were once on the White House website, were removed as is customary when a new administration takes over, but can now no longer be found on the Trump administration's post-White House website. Our team was able to copy much of the text from policy documents that have since been removed from government websites. But, it is unlikely that we were able to capture every policy document that was published at any given time. This is a challenge that any team doing this type of research would face, and it is not likely to ever be fully addressed. During the COVID-19 pandemic, there were countless governmental agencies at every level implementing policy guidance. Additionally, due to the political nature of much of the governmental responses in the U.S., many of the subnational governmental policy responses were different compared to federal government agency recommendations (e.g., some states enacted strict social distancing guidelines while others focused on keeping small businesses afloat). This inconsistency, and the time constraints of the project, led to increased difficulty for the research team, who gathered as many diverse policy documents as possible.

In our paper, we focused only on ideas and institutions since analyzing interests would require measuring public support in a way that policy document data would not permit. We do, however, return to the intersection of *ideas, institutions*, and *interests* as drivers of policy change, an intersection that was first described by Heclo (1994). Heclo offers perhaps the most concise summary of the three pillars of Policy Regime analysis available today, “Interests tell institutions what to do; institutions tell ideas how to survive; ideas tell interests what to mean.” In other words, institutions refine ideas (shared beliefs) through the interests of actors and then develop guiding principles which inform plausible policy responses. Taking the Donald Trump Administration as a proxy for interests, the Trump Administration was often combative with the leadership of federal government agencies. For example, the Administration's open dispute with the leadership of the U.S. Department of Health and Human Services, which published a report on shortages of masks and other essential safety equipment in American hospitals. Ultimately, it fell on state and local level decision-makers to make sense of the chaos and implement solutions to shortages. These solutions looked different in all 50 states. In Alabama, for instance, they relied on existing disaster plans, such as the 2009 Alabama Healthcare Disaster Planning Guide and its stockpile of personal protective equipment, including over a million surgical masks (Alabama Department of Public Health, 2009). By 2020 however, many of these stockpiled items had gone missing or were unusable, leading to local actors such as businesses improvising asking customers to use handkerchiefs or scarves as face covers.

Some of the incoherence of the federal, state, and local responses begs the question: would COVID-19 responses be more effective in a place with a centralized governmental system? Kettl (2020) notes that the decentralized initial response by the federal government led to varied state responses. Tied to these varied responses were increasing levels of friction between every level of government and that “these frictions had real impacts on the health of Americans” (Kettl, 2020). Cai et al. (2021) use the Policy Regime Framework to examine China's response to COVID-19. They found that China's response capabilities were hindered by its strict top-down governmental structure, which resulted in poor early-warning and preparedness capabilities. Furthermore, the party-state's rigid control structure incapacitated grassroots organizations and volunteers, failing to generate cohesion and interest alignments. Given the similar outcomes in the U.S. and China, differences between centralized Chinese systems or decentralized American systems provide a compelling area for future research. Carter and May (2020) provide some preliminary answers, finding that the U.S. initially displayed an incoherent response to COVID-19, which undermined the U.S.' capacity to mitigate the spread of the virus and ultimately led to impaired legitimacy and the deterioration of the federal policy regime. They also questioned the ability of the U.S. to get all states “on the same page” and foster the legitimacy and bi-partisan cohesion necessary to prevent the U.S. death toll from surpassing 136,000. At the time, Carter and May (2020) considered this number “sobering;” as of May 2022, U.S. COVID-19 deaths have reached nearly 1 million (Centers for Disease Control Prevention, 2022a), further justifying a closer examination of the U.S. response to COVID-19 using the Policy Regime Framework. Unfortunately, while we can quantify the damage COVID-19 has done in the United States, in some countries like China, it remains impossible to know COVID-19's actual cost to human life; a lack of governmental transparency has led to notoriously misleading and underreported statistics (Adam, 2022).

In our analysis, we use the concepts developed here to show how leading actors at the federal, local, and state levels aligned with *ideas* and *institutions*. There were two distinct messaging periods in the U.S. during the COVID-19 pandemic: pre and post-vaccine. We also found that there were significant differences in the ways that federal, state, and local governments approached each. Federal level *ideas* focused on messaging that communicated to the public that a public health emergency was unfolding and promoted safety actions such as social distancing and provisioning of supplies in an emergency capacity. At the state level, those federal-level ideas became concrete policy responses (from the executive branch of government, governors, or large public universities). At the local level, government entities focused on ensuring citizens understood how to stay safe through personal protective behaviors like social distancing and enforcing those desired behaviors. Federal level messaging on *institutions* focused on the White House, the Coronavirus Task Force, and the private sector as a federal partner for implementation. States' responses focused on the executive branch and its Emergency Orders. States also partnered with large institutions, such as state university systems, to implement the ideas emanating from federal sources like the CDC. Local governments focused on enforcing specific safety actions and mandates derived from federal guidelines and recommendations. The most important contribution of our research is to examine what happened in three of the lowest income states in the U.S., where people of color and other minority communities were disproportionately impacted by the pandemic. Our research shows how federal, state, and local governmental action, mainly messaging, occurred in the pre- and post-vaccine stages of the pandemic. It is imperative to take from this research the need for public health measures and future epidemic responses to be removed from political dialogue to the extent possible in the U.S.'s current political environment. In a time of increasing polarization between the two major political parties, it is necessary to ensure in the face of future epidemics that governmental responses and recommendations are made with the public's best interest in mind. It is increasingly important in marginalized communities that already experience disproportionate levels of sickness and death during a global pandemic that all levels of government coordinate and respond to public health crises in lockstep.

## Data availability statement

The raw data supporting the conclusions of this article will be made available by the authors, without undue reservation.

## Author contributions

GJ was the first author, the lead writer, and an editor throughout the research. KW was the primary editor, second author, and created the COVID timeline. KD provided most of the materials/methods and helped with editing throughout. JW wrote the R code, ran the analysis, and edited. RW and KC provided editing and writing guidance. MB and TM helped with editing. All authors contributed to the article and approved the submitted version.

## Footnotes

^1^For more examples see: “*It's going to disappear”: A timeline of Trump's claims that COVID-19 will vanish* (Wolfe and Dale, 2020).

^2^The CDC is the U.S.' major national public health agency in the Department of Health and Human Services. It is in charge of ensuring the public health of American citizens and responding to health crises.

^3^The Food and Drug Administration in the United States approves medication, tests for diseases, and vaccinations, among other responsibilities.

^4^For more examples which apply the policy regime lens see (May and Jochim, 2013, p 427).

^5^FEMA is the U.S. disaster response agency. It is in charge of coordinating disaster response and assisting state and local governments with resources and funding.

^6^State governments in the U.S. provide significant funding for state universities. This funding assists in keeping tuition costs down, allowing for more residents to enroll in university.

^7^Of these 170 data points of ideas, 103 were from federal agencies, 20 were from state agencies, 16 were from local agencies, and 31 were null.

^8^A Major Disaster Declaration is when FEMA formally declares a disaster, unlocking federal resources for subnational levels of government including cities and states.

^9^This unit was established in 2015 by the Obama administration's National Security Advisor Susan Rice (Reuters, 2020).

^10^Through tax reform in December 2017 and more proposed budget cuts in February 201.

## Appendix A

### Search Terms

Search terms to find policy documents:

- “COVID-19 Response United States of America federal government”
- “COVID-19 Response Centers for Disease Control”
- “COVID-19 Response Alabama Department of Public Health”
- “COVID-19 Response Alabama”
- “COVID-19 Response Louisiana”
- “COVID-19 Response Louisiana Department of Health”
- “COVID-19 Response Mississippi”
- “COVID-19 Response Mississippi State Department of Health”
- “COVID-19 Response Huntsville”
- “COVID-19 Response Birmingham”
- “COVID-19 Response Montgomery”
- “COVID-19 Response New Orleans”
- “COVID-19 Response Baton Rouge”
- “COVID-19 Response Shreveport”
- “COVID-19 Response Jackson”
- “COVID-19 Response Gulfport”
- “COVID-19 Response Southaven”
- “COVID-19 Response Auburn University”
- “COVID-19 Response University of Alabama”
- “COVID-19 Response Louisiana State University”
- “COVID-19 Response University of Mississisppi”
- “COVID-19 Response Missisisippi State”

## Appendix B

### Coronavirus Task Force

The team members of President Trump's Coronavirus Task Force include: (1) Dr. Birx, facilitator of the United States' participation in the Global Fund to Fight AIDS, Tuberculosis, and Malaria who would become the Task Force coordinator; (2) Alex M. Azar II, then the Secretary of HHS and appointed chair of the Task Force; (3) Dr. Robert R. Redfield, [then] director of the CDC, already in charge of overseeing the United States' response to the coronavirus; (4) Dr. Antony S. Fauci, head of the National Institute of Allergy and Infectious Diseases at the National Institutes of Health since 1984; (5) Kenneth T. Cuccinelli II, [then] leader of the United States Citizenship and Immigration Services in the Department of Homeland Security; (6) Dr. Jerome Adams, [then] United States surgeon general; and (7) Seema Verma, [then] administrator of the Centers for Medicare and Medicaid Services (Shear, 2021). Initially, the Task Force focused on keeping infected Chinese Citizens from coming to the United States while simultaneously evacuating several thousand Americans from China: “The genesis of [the Task Force] was around border control and repatriation,” said a senior official involved in the meetings.
